# Emotional experiences and coping strategies of nursing and midwifery practitioners in Ghana: a qualitative study

**DOI:** 10.1186/s12912-020-00484-0

**Published:** 2020-10-06

**Authors:** Joshua King Safo Lartey, Joseph Osafo, Johnny Andoh-Arthur, Kwaku Oppong Asante

**Affiliations:** 1 Department of Organisation and Human Resource Management, University of Ghana Business School, Accra, Ghana; 2 Department of Psychology, University of Ghana, P. O. Box LG 84, Legon, Accra, Ghana; 3 Centre for Suicide and Violnece Research- (CSVR), Ghana, Republic of Ghana; 4 Department of Mental Health, Faculty of Medicine and Health Sciences, Norwegian University of Science & Technology, Trondheim, Norway; 5 Department of Psychology, University of the Free State, Bloemfontein, South Africa

**Keywords:** Emotional regulation, Emotional labour, Emotional demands, Coping strategies, Nurses and midwives; Ghana

## Abstract

**Background:**

Emotional regulation forms an integral part of healthcare delivery. In the performance of the core duties of nursing and midwifery, health professionals are expected to enhance occupationally/organisationally required emotions. The purpose of this study is to explore.

The meaning nurses and midwives give to emotional labour as well as the coping resources employed by these professionals in order to manage the emotional demands of their profession.

**Method:**

A qualitative study was conducted using a semi-structured interview guide with fifteen (15) purposively selected nurses and midwives. Interviews were recorded and simultaneously translated and transcribed. Thematic analysis was used to analyse the data.

**Results:**

Our findings showed that participants conceptualized emotional labour as display of rules. Sadness, abuse and bullying, poor incentivisation, emotional exhaustion and emotional mix bag were reported by the participants as emotional demands and deficits. Nurses and midwives coped with emotional labour through the use of five (5) main resources: psychological capital, routinisation of emotions, religious resources, social support and job security.

**Conclusion:**

Nursing and midwifery professional duties are accompanied with emotional regulations which tend to have consequential effects on a myriad of work-related issues. Clinical healthcare training needs to intensify and equip professionals with the skills of regulating and managing their emotions since managing emotional demands are central to effective healthcare delivery.

## Background

At the heart of nursing and midwifery service delivery are emotional regulations and management [1, 2]. Thus, the professional duties of nursing and midwifery are accompanied by diverse emotional situations including, but not limited to, abuse by supervisors and clients, death of clients which elicits sadness and chronic conditions of clients which are both physically and emotionally demanding and therefore, leads to job burnout [3–5]. Even though there exist various areas of emotional regulation and management in professional healthcare, emotional labour has become one of the areas of concern in recent emotional management literature [6], especially among nurses [7]. To explain emotional labour, nurses and other health professionals, as part of their job description, are expected to show positive and organizationally/occupationally desired emotions especially, to clients [7]. Thus, the engagement in emotional labour by nurses and midwives enable them to maintain a cordial relationship with clients [5]. It must be acknowledged that nurses and midwives experience a wider range of emotional situations including anger, guilt, helplessness and others, regardless of context [3, 8].

Extant literature has identified two broad strategies employed by service workers including nurses in the process of engaging in emotional labour; surface acting and deep acting [9, 10]. That is when nurses encounter emotional situations in their line of duties, they are expected to always show enough external positive emotions such as empathy and happiness if they are internally not [7]. Surface acting has been explained as the suppression of internally felt emotions with the purpose of enhancing organizationally or occupationally desired emotions [10, 11]. On the contrary, deep acting is conceptualised as engaging in emotional expressions which are consistent with internally felt emotions [9].

There is much evidence in support of the argument that since surface acting requires suppression of internal feelings, there exists an inner tension or emotional dissonance when employees engage in surface acting [12–15]. This, in turn, has a negative bearing on employee work-related issues, including their job attitudes (job satisfaction and organisational commitment) [16]. However, the evidence in support of the consequential effects of deep acting on employee job attitudes is inconclusive [17–19]. Reasons for this have been pointed to the research approach (mostly quantitative) and context [20].

There is, therefore, a need to employ a research approach which focuses on unearthing the inconsistencies in the relationships between emotional labour and job attitudes. Additionally, empirical studies on the subject matter in the African context is at the embryonic stage with most studies tailored towards exploring the nature of emotional labour [7, 21]. In the same vein, theoretical arguments in relation to the outcomes of emotional labour are heavily based on the emotional labour theory proposed by Grandey [13]. The present study provides a new perspective and theoretical argument (job demand- resources model) to gain an understanding of the outcomes of emotional labour.

The present study is a follow-up to a previous study in Ghana that revealed that while surface acting was significantly related with job attitudes, deep acting did not [14]. Furthermore, the same study showed that perceived organisational support moderated the relationship between deep acting and job attitudes but did not play a moderating role in the relationship between surface acting and job attitudes [14]. Even though empirical studies on emotional labour is emerging especially, in the African context, a study among media practitioners in Ghana asserted that emotional labour is conceptualised as faking (pretense and emotional enhancement) [12]. The same study also reported that emotional labour (surface acting and deep acting) had an inconsistent relationship with job attitudes and psychological health [12]. Another study using hermeneutic phenomenology found that clinical learning experience among nursing students is heavily immersed with emotion-related situations and therefore, emotional labour forms a core part of nursing learning experience [7]. The same study further revealed that nursing requires engaging in varying emotions due to caring encounters, death of patients as well as caring-learning relationships expected to be harnessed during clinical nursing [7]. Due to the identified gap in literature, the current study is to explore the reasons for such discrepancies in the relationship between emotional labour and job attitudes. To achieve this, we adopted a different methodological approach-qualitative method, and employed a different theoretical argument.

### Theoretical underpinning

Grandey’s [13] conceptualization of emotional labour provides much theoretical foundation to the present study. Grandey [13] argues that in line with displaying required emotions at work, employees, including health professionals, engage in either surface acting (hiding or faking felt emotions) or deep acting (try to experience the desired emotions). The engagement in surface acting or deep acting is anticipated to have impact on employee work-related attitudes and outcomes. Besides, theoretical arguments from the job demand-resource model postulate that work environment is associated with demands which tap into the physical, emotional, psychological and other resources of the individual [22, 23]. The demanding nature of work mostly have negative consequences on work-related factors including, but not limited to, job attitudes, job performance and job outcomes [22, 23]. Employees therefore fall on diverse resources available at work in order to manage and cope with such demanding nature of work [22, 23] The availability and extent of these resources play a significant role in determining the consequences of the work demands on work-related factors including job attitudes [23, 24].

On the basis of the theoretical models, the present study was interested in exploring the following questions: 1) how do nurses and midwives conceptualize emotional labour?; 2) what are the emotional demands of their work?; and 3) what are the coping resources nurses and midwives adopt in managing the emotional demands of their work?

## Methods

### Philosophical orientation

The underlying philosophy to the present study is the interpretivism (constructivism) which emanates from the idealism perspective [25]. Although many grades of constructivism exists [26], they all share three main features postulated by Lincoln and colleagues [27], a relativist ontology a subjectivist epistemology, and naturalistic methodological procedures Relativist ontology implies there is no single reality; instead, there are multiple realities which are based on the construction and the interpretation of the realities of individuals [25, 28]. Consequentially, the interpretation and construction of issues are based on a co-creation of knowledge attainable through researcher-researched cooperation. That is, ‘truth’ is subjective and therefore, multiple. Hence, knowledge is generated through the interaction between a researcher and an interviewee. Hence, situating studies within naturalistic setting brings the context to bear in the generated knowledge) [25, 27].

### Research design

The methodology used in the present study should be understood as a sequence and a follow-up to an earlier study see [14]. The study employed an explorative and a multiple case study design with the purpose of unravelling the nuisances and contradictions that emerged in the earlier quantitative study [25]. The method for the present study as indicated earlier is qualitative. It is essentially concerned with meaning and generating new insights into old problems [29, 30].

### Population, sample and sampling technique

The population for the qualitative study involved staff nurses and midwives, including their managers, in six public hospitals in the Greater Accra region of Ghana. The staff nurses and midwives of the various units, departments and health facilities were chosen for this part of the study because they have been engaged in the health care profession for a considerable number of years and have diversified experiences in relation to the emotional demands of health care. In a qualitative study, there is an attempt to understand a smaller number of participants’ worldview rather than testing hypotheses based on a large sample [31]. Accordingly, a sample size of 15 was selected for the study. In accordance with the reflections of Baker and Edwards [32], a sample size of 12–60 is adequate to thoroughly explore a phenomenon qualitatively. We were further guided by the principle of saturation where we terminated data collection when we were not finding any additional information from successive interviews [31, 32].

### Inclusion and exclusion criteria

In line with the earlier study, only staff nurses and midwives with not less than 6 months of work experience were targeted for the study. This criterion was used because job attitudes are developed over time after working with an organisation [33]. As well, only nurses and midwives who were employed by the Ghana Health Service formed part of the study for the purpose of homogeneity. Hence, student nurses and midwives, as well as rotation nurses and nurses with less than 6 months of working experience were excluded from the study. Further, respondents who were part of the first phase of the study (quantitative study) served as another criterion for selection. In addition, all nurses and midwives who were not part of the prior study and/or have less than six-month working experience were excluded from the qualitative study. A summary of the demographic characteristics of the sample used is presented in Table 1.

**Table 1:** Demographic characteristics of qualitative study participants

Code	Age	Marital status	Religion	Position	Job title	Cadre of nurse	Years of practice
A	35–39 years	Married	Christian	Managerial	Principal nursing officer	General nurse	10 years
B	30–34 years	Married	Christian	Non-managerial	Nursing officer	Psychiatric Nurse	7 years
C	30–34 years	Married	Christian	Managerial	Nursing officer	General nurse	8 years
D	55–59 years	Married	Christian	Managerial	Deputy director of nursing	Psychiatric nurse	33 years
E	30–34 years	Married	Christian	Managerial	Nursing officer	General Nurse	8 years
F	35–39 years	Married	Christian	Managerial	Nursing officer	General nurse	11 years
G	25–29 years	Married	Muslim	Non-managerial	Nursing officer	General Nurse	5 years
H	25–29 years	Single	Christian	Managerial	Nursing officer	General nurse	5 years
I	55–59 years	Married	Christian	Managerial	Principal nursing officer	General nurse	37 years
J	55–59 years	Married	Muslim	Managerial	Midwife	Midwife	23 years
K	30–34 years	Married	Christian	Non-managerial	Midwife	Midwife	7 years
L	30–34 years	Married	Christian	Managerial	Nursing officer	Psychiatric nurse	9 years
M	30–34 years	Married	Christian	Managerial	Senior nurse officer	Psychiatric nurse	7 years
N	55–59 years	Separated	Christian	Managerial	Principal nursing officer	General nurse	36 years
O	30–39 years	Single	Christian	Non-managerial	Nursing officer	General nurse	9 years

### Semi-structured interviews

The study used a semi-structured interview guide which was carved out of the results from the earlier. The interview guide was divided into four main sections. The first section focused on the demographic characteristics of the interviewees, including gender, age, job title, job duties, and tenure of work. In the second section, the guide focused on the meaning interviewees gave to emotional labour. For example, “*does your professional duties require you to engage in certain emotions*?”. The third section of the interview guide elicited the emotional demands of nursing and midwifery. Typical example of a question on the guide was “*is your profession emotionally demanding? Can you share some of them with me?”*. In the final section, the researchers focused on unearthing coping mechanisms used by nurses and midwives to explain the link between emotional labour and job attitudes. Examples of question on this section are “*how are you able to handle these emotional demands of your profession*” and “*do you think engaging in these emotional requirements of your profession can affect your job attitudes?”* (see additional file 1 for the interview guide). Data was collected via face-to-face interviews with respondents which were recorded on tapes.

### Data collection procedure

Data collection began with seeking ethical approval from the Ethical Committee of the Department of Organisation and Human Resource Management at the University of Ghana Business School and the various health facilities where the study was conducted. The ethical approval paved the way to contact potential respondents and their consent sought. The purpose of the study was thoroughly explained to potential interviewees. As well, the confidentiality and anonymity of interviewees were assured to them ahead of consenting to the study. Interviewees consented to the interviews by pending their signatures to consent forms. All interviews were conducted face-to-face at the health facilities with nurses and midwives who met the study’s inclusion criteria. The flexibility of the semi-structured interview, which was employed allowed the researchers to dig deeper into the issues of concern. Therefore, where necessary, follow-up questions were asked to gain further information. All face-to-face interviews were recorded on a recorder with each interview lasting between 30 and 45 min.

### Data analysis

Audio-recorded interviews were transcribed verbatim and manually analyzed using [34, 35] six phases of thematic analysis. The process included (1) familiarisation with the data; (2) generating initial codes; (3) searching for themes; (4) reviewing themes; (5) defining and naming themes; and (6) producing the report. The first step was to transcribe the data verbatimsince all the interviews were conducted in English language. The second stage was the iterative process (where the researchers read and reread the data and got deep into the data). In the third and fourth stages, the researchers identified themes and codes relevant to the current research’s aims and objectives. In the fifth stage, identified themes were restructured and revisited to ensure that the analysed data were focused and detailed enough. The final step was where the coded statements were then grouped under different broad themes. Following recommendation on improving the validity of qualitative research, we made attempts to capture exactly what participants intended (participant validation) by obtaining feedback from them [36]. Further, each stage of the analysis intensely engaged co-authors discussing initial themes until we reached consensus. Such cross-validation and group interpretation are useful in increasing inter-subjective comprehension, analytic rigour, and validity of the interpretations of the findings [37, 38]. In order to hide the identities of the participants, we used pseudo-names to participants narratives for anonymity purposes. .

## Results

The significant relationships between surface acting and job attitudes but no significant relationship between deep acting and job attitudes as witnessed by [14] created a clear path for the qualitative study. In the qualitative study, the researchers were interested in gaining an understanding of how the respondents conceptualised emotional labour, unearthing the emotional demands of healthcare among nurses and midwives and the underlining reason(s) which accounted for nature of the relationship between emotional labour and job attitudes. A summary of the themes and sub-themes which emerged from the qualitative data analysed is also presented in Table 2.

**Table 2:** A summary of the thematic analysis of the qualitative data

Themes	Sub-themes	*Selected quotes*
Conceptualising emotional labour	•Display rules	for example, when a patient or a relative makes you angry or may be verbally he abuses you or something, there even though displaying emotions is part of you, you are not expected to display that negative emotion. So when it comes to those negative emotions like attack and things you are expected to control yourself and not to play along these negative emotions. (A: 09) *So you are always required to show happiness, no sign of remorse, no sign of stress, tension, no-no, you are always supposed to be happy before your clients because we believe that a happy emotions promote healing so you are always supposed to show this uhm, create that atmosphere around you so that your patient get this motivation that he is getting well. (E: 06)*
Emotional demands and deficits	•sadness •Emotional exhaustion •Abuse and bullying •Emotional mix bag •Poor incentivization	yeah, so many. Come to think about the conditions we go through, people losing their loved ones and you see some parents acting in quote irresponsibly, may be the child is sick, keeping the child in the house and reporting in the last minute. All these things, you know, make us to be very emotional and more especially, when you also think that because of some irresponsibility on the side of parents, they leave their children to die through accidents, drinking poison, falling into wells and those things, it also adds up. And then some parents, even though the national health insurance for children is free, they don’t take advantage of it, they come here, they don’t even have money to do registration, when you look at all those things they can make emotional about it (A: 07). *The demand is very high, sometimes is so stressful that you can’t even take it. You just go like what, a man doing this, something that, like, as a man when you do those kinds of job, you feel so down, you feel demoralize, you doing those kind of work, it’s like let me even put it in a quote you are wearing a vagina, you feel like you are a woman, you have been dejected, that’s the, it’s beyond explanation emotionally, you feel so down because even with masks, those who defecate on themselves, even with mask, you can still inhale it, it’s not easy at all and you have to clean the person to look very-very neat and pleasant (H: 03)* *oh, I quite remember one time a patient poured water, bucket full of water on a staff claiming that the staff looks like “shrek”, she watched some cartoon, but I don’t know the mood of the staff but then she wept, how can you compared me to shrek (M: 09*) *Yes, at times you smile, you can cry. When you see that what a client is doing is not right or something. I quite remember one time a client came in, she is very beautiful, very educated. When she came here, she was in a mess so I took her to the washroom, I bathed her, I dressed her and then she came back to normal and then I told her you are very-very beautiful but in all things remember to give thanks to God. I started crying when I was talking to her, in fact I cried, seeing such a person in this condition. Then some too, when they crack jokes, you smile with them and then you also crack jokes especially, to those who are moody, you do certain things for them to be happy, you do certain things for them (B: 04)* *.So for instance, if the motivation is low I have to be looking elsewhere. When I’m talking about motivation not just salary but maybe something small-small. If you do something and you are acknowledged that ooh you are doing well, keep it up, then I will be committed in doing more. But when the situation is like you are doing something and nobody is even appreciating you then I don’t feel the motivation, it means I won’t do it (K: 12).*
Coping with emotional labour	•Psychological capital •Normalization/routinization	*yeah, (laughs) no human being is the same, we are all special in our own way therefore I think I’m special that’s how I’m able to handle these demands. I’m able to stand a lot of pressure even with patients who are uh, difficult. (F: 13)* *You don’t take it personal because if you do you will die early, oh yes, you don’t take it personal because somebody can just come and stand in front of you and insult you without any cause. You are consulting with doctors and because they think you are consulting with doctors, you can discharge them so after the prescribers have left, they will come to you, I want you to discharge me and you will tell them you cannot, eeii, it’s a different issue altogether. So I think we’ve grown to accept, like it’s the nature of the job so we don’t really take them personal (O:09)*
	•Religion/spirituality •Social support	for me, I see this profession as a calling so I do the work wholeheartedly. My Bible teaches me to love my neighbor as myself so I try as much as possible to understand and emphatise with other people. (C: 11) *Ohh, as for family they are always encouraging us. As for my family, they are always by my side and they encourage me a lot especially my husband and my children. After all, how many years is left, you will soon go on pension and I’m always happy for that (I: 14)*
	•Job security	*Because at the end of the day you know that at the end of the month you will receive something, it’s better than you not working at all but if there is any other chance, any moment from now, it’s just that they are just keeping me, I want to move, I want to divert totally (L: 12)*

### Conceptualizing emotional labour

#### Display rules

One of the major themes which emanated from the qualitative study, as indicated in Table 2, was conceptualising emotional labour. Even though the concept of emotional labour is relatively new among the nurses and midwives, their interpretations and meanings of situations reflect display rules. Thus, they viewed emotional labour as part of their job duties; that, it is expected of them to engage in organizationally/occupationally desired emotions which require them to either enhance positive emotions or suppress negative emotions. The quotes illustrate this perspective*:*

*Well, it is expected of me to be myself when I’m on duty, not try to pretend, you understand. So, I need to feel relaxed in my working environment and be myself because come to think of it, you spend most of your hours here so if you come here and you are going to be acting throughout then you yourself you will not be satisfied. So, it is expected that as I come to work, I need to work in a relaxed environment. Since I’m the one delivering the service it is expected that I’m relaxed and in my best frame of mind and I can give my best without any compulsion or persuasion. That’s how I feel about it* (D: 04).

The curative capital for patients with such emotional regulation is deeply expressed by another participant who has worked for 8 years. As indicated below, the health worker’s emotion is client-tethered in line with organizational expectations. It is an intentional emotional regulation that is aimed at promoting the wellbeing of the patient rather than that of the health worker:

*So, you are always required to show happiness, no sign of remorse, no sign of stress, tension, no-no, you are always supposed to be happy before your clients because we believe that happy emotions promote healing, so you are always supposed to create that atmosphere around you so that your patient get this motivation that he is getting well.* (E: 06).

Negative emotion is thus normatively prohibited. The participant below establishes a connection from a cultural norm of emotional regulation to explain her current expected organisational emotional behaviour. Her explanation reveals that the expected organizational emotional behavior might be accentuated by a cultural socialisation that is proscriptive of expressing negative emotions:

*For example, when a patient or a relative makes you angry or may be verbally he abuses you or something, there even though displaying emotions is part of you (*sic*), you are not expected to display that negative emotion. So, when it comes to those negative emotions like attack and things you are expected to control yourself and not to play along these negative emotions* (A: 09).

### Emotional demands and deficits

Aside the meaning that nurses and midwives gave to emotional labour, they also asserted that they encounter a myriad of emotional situations in the course of their professional duties. The emotional demands as reported by these health professionals range from positive emotions (happiness) to negative emotions (sadness, abuse, and emotional exhaustion). However, the emotional demands of healthcare, as alluded to by nurses and midwives, are not compensated for (emotionally) by superiors and organisations.

#### Sadness

Healthcare delivery among nurses and midwives is accompanied with emotional situations which hover around sadness. This is mostly true because some patients are lost (die) in the process of receiving healthcare. Emotional attachment nurses and midwives develop toward their clients makes the loss of patients become a more saddened situation. However, these health professionals are not expected to show sadness as a professional emotional expression. In relation to this, one of the nurses assert that:

“*Yeah, so many. Come to think about the conditions we go through, people losing their loved ones and you see some parents acting in quote irresponsibly, may be the child is sick, keeping the child in the house and reporting in the last minute. All these things, you know, make us to be very emotional and more especially, when you also think that because of some irresponsibility on the side of parents, they leave their children to die through accidents, drinking poison, falling into wells and those things, it also adds up”* (A: 07).

#### Abuse and bullying

Another emotion demands of nursing and midwifery is emotional and physical abuse. The abusive nature of healthcare is mostly experienced by psychiatric nurses due to the kind of patients they take care of. The abusive nature of caring for patients is expressed by one psychiatric nurse as:

“*oh, I quite remember one time a patient poured water, bucket full of water on a staff claiming that the staff looks like “shrek”, she watched some cartoon, but I don’t know the mood of the staff but then she wept, how can you compare me to shrek”* (M: 09).

A similar situation is re-echoed by another nurse as:

*“Sometimes you the staff too, how the client comes with the symptoms, you will be insulted from head to toe (laughs), they will make you less. Sometimes if it’s not the work per say you will do something that later you will regret it, you have to hold on to your anger. So those are some of the emotional things that we go through”* (D: 03).

Some nurses also reported bullying from superiors. The narrative below is an illustration:

*May be there is an issue, we expect them to may be come to the ground and investigate or try to understand why this happened, but you will just be there, and they will just call for disciplinary committee, you will just go and face it, (laughs), they don’t just come down to really understand what is really on the ground because working here and there, it’s two different things altogether* (O: 11) .

#### Poor incentivization

Employees generally, expect their organisations to show much concern about their wellbeing and also value their contribution. The absence of organisational support makes employees feel the negative impact of the emotional demands of their profession and are therefore, riskier to engage in negative job attitudes. On the contrary, the availability of support from organisations serve as protective factor against the negative impact of emotional demands. However, nurses and midwives in the Ghanaian context perceive their organisations /employers to be less supporting. One of the midwives attested to this:

*..So, for instance, if the motivation is low I have to be looking elsewhere. When I’m talking about motivation not just salary but maybe something small-small. If you do something and you are acknowledged that ooh you are doing well, keep it up, then I will be committed in doing more. But when the situation is like you are doing something and nobody is even appreciating you then I don’t feel the motivation, it means I won’t do it* (K: 12).

Incentivizing through social reinforcement seems to be what the participant indicates as ‘something small-small’. She seems to place this on the same weighting scale as salary. She finds the lack of such emotional support as demotivating. To further substantiate the assertion, a general nurse alluded that:

*As for me, my life is, when I do something I want to be appreciated so if I’m not appreciated on what I’m doing I don’t see the need to continue. I have to go elsewhere where I will be appreciated for my job and then my output. Because if you are appreciated you will be doing more, you understand. When we were kids and you go to exam and they write 100% keep it up, aren’t you motivated to do more next time? But when even the teacher is beating you, then you are like eii, this teacher “paa”, then I will fail for you to beat me. So that’s how it is* (M: 12).

#### Emotional exhaustion

Healthcare is noted to be stressful due to varying health conditions presented to professional workers. The stressful nature of healthcare professional duties tends to have a consequential effect on emotional wellbeing of the healthcare workers such that they get emotionally exhausted. In relation to this subject, one of the nurses who happens to be a male shared that:

“*The demand is very high, sometimes is so stressful that you can’t even take it (*sic*). You just go like what, a man doing this, something that like, as a man when you do those kinds of job, you feel so down, you feel demoralized, you doing those kind of work, it’s like, let me even put it in a quote, you are wearing a vagina, you feel like you are a woman, you have been dejected,, it’s beyond explanation emotionally, you feel so down because even with masks, those who defecate on themselves, you can still inhale it, it’s not easy at all and you have to clean the person to look very-very neat and pleasant* (H: 03).

The participant above communicates feelings of exhaustion arising from an experience of working on a task considered a stereotypical female job in the society and even at the workplace. For example, cleaning a pregnant woman and other related issues. Given the patriarchy in Ghana, it is possible that merely working in a stereotypical female profession (e.g. nursing) as a man may in itself attract some opprobrium from segment of the society. However, for the participant above, it appears the task of caring for patients including cleaning patients up and dressing them was not only overwhelming but also denigrating in his worldview.

#### Emotional mix bag

Even though healthcare is accompanied with negative emotional situations, it cannot be overlooked that there are situations which connote a bouquet of emotions (both positive and negative). Healthcare delivery among nurses and midwives presents itself with a bouquet of emotions that are both positive and negative in nature. The following quotes illustrate the emotional mix bag nature of healthcare delivery which are alluded to by nurses and midwives:

*Yes, at times you smile, you can cry. When you see that what a client is doing is not right or something. I quite remember one time a client came in, she is very beautiful, very educated. When she came here, she was in a mess, so I took her to the washroom, I bathed her, I dressed her and then she came back to normal and then I told her you are very-very beautiful but in all things remember to give thanks to God. I started crying when I was talking to her, in fact I cried, seeing such a person in this condition. Then some too, when they crack jokes, you smile with them and then you also crack jokes especially, to those who are moody, you do certain things for them to be happy, you do certain things for them* (B: 04).

“*For example, when a patient looks that bad and you think that this one 50-50 and you see the patient recovering, the patient is talking back with you, you are happy, you are this and then you even share with them, wow, you scared me to death, I was so much afraid. You know, those things, they help you to know that you are doing the work whole heartedly or even if you cry with the patient when they lose their loved one, they will know that oh, even the nurse cried, they will also know that you’ve done your best, you feel very sorry that you couldn’t save that love one for them and at the end of the day they also don’t blame you for what has happened because you also showing some emotions which means that you are also a human being, you can also be in the same soup, that is also what releases comfort in me”* (A: 05).

### Coping with emotional labour

Even though engaging in emotional labour mostly gives birth to negative work attitudes and outcomes, employees over time develop coping mechanisms or fall on some resources in order to deal with the emotional demands of their profession. These included *psychological capital, routinisation/normalization, religious resources, social support and job security*.

#### Psychological capital

These encompass all individual, intrapersonal unique qualities which enable nurses and midwives to deal with the emotional requirements of the profession. The quotes below substantiate this:

*yeah, (laughs) no human being is the same, we are all special in our own way therefore I think I’m special that’s how I’m able to handle these demands. I’m able to stand a lot of pressure even with patients who are uh, difficult* … …. (F: 13).

Others explained their psychological capital along temperamental and religious lines. The participant below measures her personal resource in her ability to effortlessly, confront others for a perceived injustice, forgive, and repair the relationship. It seems that she connects this aspect of her to her religious faith:

*I’m free, I’m very free, when you do something to me, I will tell you that day, that place, finish! I don’t send it home, ask them they will tell you. I’m free, if you do me something I won’t send it home oo, I will just tell you this is what you’ve done to me, I don’t like it and there it is finished! Somebody will see me the following day and not greet me. I will call you, come here, why are you not talking to me just because of yesterday? Are you a Christian or a Muslim, when you went and slept, did you pray?* (J: 12)*.*

A participant reported the regulation of her emotion in a more mechanical fashion: that she could turn it on and off. Her emotional regulation is thus very intentional and agentic: *Me, I’m able to put my emotions in check so I decide when I want to get angry then I get angry, I don’t just get angry* (C: 08).

#### Routinization/normalization

Thus, nurses and midwives mostly see the emotional demands of their work as normal and routine aspects of the profession. The normalization and/or routinization of their emotional requirements enables nurses and midwives to handle and manage the emotional circumstances of their profession. In the words of a female general nurse with a 11-year work experience, she alluded that:

*Errr, it has become a routine thing so as and when it comes I handle it but I try very hard, yes, I try very hard to keep more especially, negative emotions under control but for the positive ones, I try to display them because I want my patient to see me outside and say that this nurse helped me a lot, this nurse is really good* (A: 10).

As indicated above, although emotional regulation is an integral part of her work, she reports that the negative aspects are difficult to manage. Her patient-orientation seems to be a driving force.

A psychiatric nurse who has been in the profession for 8 years also attested to idea of routinization/normalization and expressed below:

*You don’t take it personal because if you do, you will die early. Ooh yes, you don’t take it personal because somebody can just come and stand in front of you and insult you without any cause. You are consulting with doctors and because they think you are consulting with doctors you can discharge them so after the prescribers have left, they will come to you, I want, you to discharge me and you will tell them you cannot. Eeii, it’s a different issue altogether. So, I think we’ve grown to accept, like it’s the nature of the job so we don’t really take them personal* (O: 09).

As can be gleaned from the above, the participant de-privatizes mistreatment from clients as means of managing her emotions. She consigns clients’ misbehavior toward her to the realm of occupational hazards. Importantly, she indicates that ‘we’ve grown to accept’ as potentially intimating that emotional regulation can be a by-product of certain organisational cultural expectations.

#### Religious resources

these sub-theme addresses all religious resources these health professionals rely on in attempt to manage the emotional circumstances of the profession. The worldview of the Ghanaian is greatly infused with religious beliefs and/or spirituality which in recent times are accentuated by Pentecostal beliefs [39, 40]. Religious or spiritual resources enable nurses and midwives to manage the emotional circumstances of their professional duties. The quotes below demonstrate this:

*Well, a lot of faith take place, I’m telling you, and prayer. A lot of faith because if you want to deal with this on a mere physical level, there will be a time you can’t continue, you will feel it’s a bother. And also, passion for the job, if nursing is not your field and you come in to make money or look for position then you will be so disappointed and so frustrated and that’s where you will end up I mean doing displacement. You will end up displacing all your anger on your client. So it’s really an important thing and so that’s what help us to manage some of these situations* (F: 14).

Faith and prayer are indicated as spiritual resources that mitigate the stressors related to emotional regulation. Accordingly, she views the nursing profession as requiring commitment that transcends personal ambition. It is in the light of the above that others reported viewing the profession as a ‘calling’ and one whose ethos seem to find a strong validation within the Judeo-Christian tradition. The participant below theologizes the profession and centralizes love and empathy as defining virtues.

*For me, I see this profession as a calling, so I do the work wholeheartedly. My Bible teaches me to love my neighbor as myself, so I try as much as possible to understand and emphatise with other people.* (C: 11).

Explicitly, others have accepted the professional demands and the emotional hazards that come along with it as a means to an end- to solicit divine benefits. Such transcendental posturing reinforces a service-oriented attitude and organisational commitment:

*Me, for instance, one thing about me is I always tell myself and my colleagues, I don’t do the work for man per say but I do it to please God, so ask them, when I come I do my best because at the end of the day God will reward me, not man because here whatever you do they don’t appreciate it, but how I have made my mind, if I rely on them it’s not encouraging to me to be committed to the organisation* (L: 10).

#### Social support

Nurses and midwives rely on support from co-workers, family, friends and significant others to enable them to better handle the emotional requirements of healthcare delivery. The extent, and richness of the social support (either perceived or received) determine the impact of the emotional demands of healthcare delivery on job attitudes. As evidenced in the quotes below, nurses and midwives greatly rely on social support in the course of performing their professional duties:

*Anyway, I think among us, we try to encourage each other … ……… … oh, for instance, when the water was poured, we had to caution the client who did that and we all came together to do that. So you’ll realise that later she came to apologise so it’s like we are all onboard together* (L: 13).

As indicated below, the management of relationships- both with superiors and subordinates is an integral part of the support system for the health workers. Further, incentives are perceived as important features of the support from superiors. Generally, these relationships are presented as ‘primary’ within the purview of organisational support:

*Sometimes from colleagues. That’s why I say when you are working with colleagues and you are working as a team sometimes you encourage one another. Even if you are in charge and you are the leader you have to be in relationship with your subordinates so that they can come to you and freely share their problems with you, then when you also have an issue, they can also support or encourage you. So sometimes support from one another as colleagues really help and when you have superiors who will also give you a listening ear and try to, if not provide all the incentive packages you need, try to give some little incentive here and there, it helps* (O: 14).

Beyond the social support which emanates from the colleagues and dynamic interactions between superiors and subordinates, participants also reported receiving support from their families:

*yeah, from my family because that’s the only place I’m able to express myself about what happened to me at work and they tell me it’s normal, that’s how your work is, try to reassure you that your reward is in heaven, that alone is satisfactory, you just get satisfied that you will get some special reward somewhere* (C: 13).

We can deduct from the above narrative that the participant sees the family as ‘containers’ to dump all her emotional stressors. The support from the family is partly rationalizing the demands of her work and also inspiring her by virtually sacralizing her profession.

#### Job security

Apart from the personal and religio-social resources which nurses and midwives harness to manage the emotional demands of their profession, they again look up to secured nature of the job. Nurses and midwives in Ghana perceive their job as a secured profession compared to others who are not employed. Most of these health professionals are assured of employment after completing their process of training and will hardly lose their jobs. The assurance of job security keeps them going in their professional duties. This is evidenced in the following quotes as proclaimed by some nurses:

“*as for that no, I can’t even think of any, the only support is that at least I’m employed, you know Ghana here most people are unemployed so if I’m employed that’s okay, I don’t want to lose my job so honestly, I don’t receive any other support* (O: 16).

This point above is corroborated by another from a different nurse below.

*Because at the end of the day you know that at the end of the month you will receive something, it’s better than you not working at all but if there is any other chance, any moment from now, it’s just that they are just keeping me, I want to move, I want to divert totally* (L: 12).

## Discussion

As witnessed from the thematic analysis, the health professionals’ conceptions of emotional labour reflects a response to normative organisational standards. Thus, nurses and midwives perceive that as part of their professional duties, it is expected of them to show certain emotions while suppressing other emotions. In line with the conceptualization of emotional labour by Hocshchild [10, 11] and Theodosius [41], emotional labour denotes the engagement in display rules by either suppressing emotions or naturally experiencing emotions. Nevertheless, the finding from the present study contradicts the finding by [12] who disclosed that emotional labour among media practitioners is conceptualised as faking. Unlike the study by [12], the professional duty of healthcare cannot encourage faking emotions because it deals with handling human lives and therefore, these health professionals (nurses and midwives) do not perceive their emotional labouring as faking but rather a response to normative organisational standards. In line with Grandey’s conceptualization and theoritisation of emotional labour, employees engage in emotional labour as a response to organizationally or occupationally desired emotions (display rules).

Also, the discovery from the present study that nursing and midwifery require engaging in a bouquet of emotions ranging from negative to positive emotions is not out of place; it corroborates extant literature. For instance, empirical studies by [3, 4, 7] point to the fact that nursing and midwifery involve diverse emotional demands including abuse from clients, supervisors and colleagues, work burnout, guilt, anger, chronic conditions of clients and death of clients. The consequential effects of negative and positive emotional experiences of healthcare professionals are established in the extant literature see [14].

In line with the economic context of Ghana, nurses and midwives perceive their profession as a source of job security because there are less alternative job opportunities. Thus, some individuals in this context, may join the profession not necessarily because of the love of the job but because there are less alternatives. This orientation by some nurses and midwives is more likely to make them prone to surface acting rather than deep acting, as disclosed in the earlier study see [14].

The present study also disclosed routinization or normalization another coping resource to emotional laborung. Thus, the routinization and normalization of the emotional experiences of health professionals tend to cushion them against the impact of the emotional demands of healthcare profession.

Furtherance to the above argument, the study also disclosed other coping resources employed by nurses and midwives to manage the emotional demands of their profession. As evidenced, these health professionals heavily draw on personal (psychological capital), religious/spiritual, social and job resources to enable them deal with the emotional demands of their profession. Thus, the extent to which these are made available to these health professionals tend to serve as a protective factor against experiencing the negative impact of the profession’s emotional demands.

Situating the findings to the job demand-resources model, professional healthcare delivery is accompanied with varying emotional experiences including, but not limited to, bullying, abuse, emotional exhaustion and sadness. Nevertheless, healthcare professionals fall on varying resources at workplace (be it personal, social, organsiational or religious) to enable them manage the emotional demands and experiences of their professional duties. Thus, findings from the present study align with the job demand-resources model.

### Limitation of the study

Even though the study employs a unique methodological approach to the study of emotional labour, the researchers acknowledge some potential flaws to the approach employed. The study design was based on a cross-sectional one which disregard changing trend over time as used in longitudinal study. A longitudinal study, if used, may also indicate some possible changes in the variables studied and give a more precise association between them. Nevertheless, the use of a qualitative method as a follow-up to the earlier quantitative study gave an insight into the variables studied.

### Implications and recommendations

Healthcare delivery is anchored with emotional regulations which are expected to conform with organizationally or occupationally desired emotional display. Nurses and midwives are always expected to show positive emotions such as showing empathy and happiness on their job. Nevertheless, the display of positive emotions by these health professionals may be consistent or contradict their inner feelings which will in turn influence their job attitudes. As witnessed in this study, the engagement in emotional labour is accompanied by varying emotional experiences. Hence, nurses and midwives, as part of their training, should be socialized to develop the necessary emotional intelligence that will enable them to manage the emotional experiences and demands of their professional duties. The development of emotional intelligence by these health professionals will sharpen and strengthen their psychological capital in the event of managing the emotional and stressful nature of their jobs.

The study also provided much evidence in support of the assertion that nurses and midwives rely on myriad resources to enable them to handle the emotional demands of their jobs. In regard, health institutions should be more willing to provide all necessary supports (physical, emotional, social, mental and others) to its employees especially, nurses and midwives since these have been found to buffer and protect employees against experiencing the negative impacts of their work duties. Further, the work environment of these nurses and midwives should be made friendlier such that coworkers, supervisors and management are able to provide social support to employees to enable them to better manage the emotional demands of their profession.

In the same vein, since the average Ghanaian is religious and/or spiritual, the professional training of nurses and midwives should acknowledge that healthcare delivery is a moral issue and should therefore, infuse religious and/or spiritual dimension. The moral and religious fortitude of these health professionals, when awaken, will shield them against the negative consequences of the emotional demands of healthcare delivery.

## Conclusion

The nature of work among nurses and midwives requires them to have an ability of managing and regulating their emotions. Their display of emotions is expected to be in line with organizationally desired emotions which also have consequences on a myriad of issues including their job attitudes. The present study has provided much evidence in support of the fact that professional healthcare is associated with emotional experiences ranging from abuse, bullying, sadness and emotional exhaustion. In addition to the above, the study found a number of coping resources employed by nurses and midwives in managing the emotional demands of the profession. The use of a qualitative research methodology to the study of emotional labour and gain better understanding of the phenomenon is evidenced in the present study.

## Supplementary information

**Additional file 1.**

## Data Availability

Data is available upon reasonable request from the first and corresponding authors.
