# Supplementary material for: Emotional experiences and coping strategies of nursing and midwifery practitioners in Ghana: a qualitative study
Source: BMC Nurs. 2020 Oct 6;19:92. doi: 10.1186/s12912-020-00484-0 (PMC7541208; doi:10.1186/s12912-020-00484-0)
Supplement: Supplementary file 1 — Additional file 1. [file 12912_2020_484_MOESM1_ESM.docx]

**INTERVIEW GUIDE**

A brief description of the research topic and main objectives will be given to intended respondents.

*This research proposal is focused on the interactions between health professionals (nurses and midwifes) and their clients. I will be asking questions about how you have to manage your emotions when dealing with these clients in your day to day work, the types of emotions that are directed at you from them and what you may do as a result of this. I will also be covering issues relating to how this emotional interaction impacts on you/your job attitudes and the type of things that help or hinder your ability to cope with the emotional demands of your job.* *Do you have any questions?*

**Personal Data / General Questions**

1. What is your Job Title/Grade?
2. How long have you been working as a health professional?
3. What does your job entail?
4. Age or age range
5. Religious Affiliation

**Emotional Displays**

1. Tell me the emotional displays you sometimes experience as a health professional.
2. Can you tell me about a time when you had to manage your emotion as part of your role as a nurse or midwife?
3. Tell me how you know which emotional displays are acceptable in your role
4. How do you make sure you show these emotions? (deliberately elicit these emotions? or naturally what they feel?)
5. Can you describe a moment or a time when you felt uncomfortable or constrained by the requirement to display these kinds of emotions?

**Emotional Constraints**

1. Tell me about the emotions you are expected to inhibit or constrain as part of your role as a nurse or midwife.
2. How do you know which emotional displays are unacceptable?
3. How do you make sure you don’t show these emotions? (Hide these emotions or hide how they naturally feel?
4. Tell me the times you feel uncomfortable not being able to display these emotions
5. How much do you consider this emotional constraint to be part of your job?
6. What causes you to engage in these emotions?

**Feedback of Responses from Clients (emotional intelligence)**

1. What sort of emotional responses do you get from the clients you work with?

- Is there a recent example you can use by way of illustration?
- How did that make you feel?

How did you manage it? (Probe for social skills, emotional management skills, self-management, empathy)

1. What are some emotional aspects of your work that makes your job as a nurse or midwife enjoyable?

**Individual Abilities**

- How do you manage/handle the emotional demands?
- What personal quality(ies) allows you to deal with these work pressures?

**Coping Strategies and Available Support**

1. **-** How do you deal with issues from clients that tend to upset you?

- Tell me the type of things that help you cope with the emotional demands of your work.
- Do you receive support from your organization that help you handle these day-to-day emotional management? Probe further (for what kind support)
- In your view, is the organizational support adequate? Probe further
- Are there any other support in managing your emotions? Probe Further
- Are there some things that reduces your ability to cope with these emotional challenges? Probe further

**Job attitudes**


- Tell me what sustains you to keep working in this industry.
- Do you see yourself working in this industry in the next 2, 5, 10, ……. years? (Probe for why)
- What personal/organizational characteristics do you think would make someone more likely to be successful in respect to emotional management aspect of your role? Probe further.
- Do you think new employees need to be given training on emotional labour and emotional intelligence? Probe further

**Concluding Questions**

1. Let us summarize the main points of our discussions (The researcher presents a review of the responses to the key questions). Is this summary complete? Are there any additions or changes you would like to make?
2. Are there any other questions you wanted me to ask you?
3. How did you feel being interviewed about this topic?
